# Drivers of the Australian Health System towards Health Care for All: A Scoping Review and Qualitative Synthesis

**DOI:** 10.1155/2023/6648138

**Published:** 2023-10-20

**Authors:** Resham B. Khatri, Yibeltal Assefa

**Affiliations:** ^1^Health Social Science and Development Research Institute, Kathmandu, Nepal; ^2^School of Public Health, Faculty of Medicine, University of Queensland, Brisbane, Australia

## Abstract

**Background:**

Australia has made significant progress towards universal access to primary health care (PHC) services. However, disparities in the utilisation of health services and health status remain challenges in achieving the global target of universal health coverage (UHC). This scoping review aimed at synthesizing the drivers of PHC services towards UHC in Australia.

**Methods:**

We conducted a scoping review of the literature published from 1 January 2010 to 30 July 2021 in three databases: PubMed, Scopus, and Embase. Search terms were identified under four themes: health services, Australia, UHC, and successes or challenges. Data were analysed using an inductive thematic analysis approach. Drivers (facilitators and barriers) of PHC services were explained by employing a multilevel framework that included the proximal level (at the level of users and providers), intermediate level (organisational and community level), and distal level (macrosystem or distal/structural level).

**Results:**

A total of 114 studies were included in the review. Australia has recorded several successes in increased utilisation of PHC services, resulting in an overall improvement in health status. However, challenges remain in poor access and high unmet needs of health services among disadvantaged/priority populations (e.g., immigrants and Indigenous groups), those with chronic illnesses (multiple chronic conditions), and those living in rural and remote areas. Several drivers have contributed in access to and utilisation of health services (especially among priority populations)operating at multilevel health systems, such as proximal level drivers (health literacy, users' language, access to health facilities, providers' behaviours, quantity and competency of health workforce, and service provision at health facilities), intermediate drivers (community engagement, health programs, planning and monitoring, and funding), and distal (structural) drivers (socioeconomic disparities and discriminations).

**Conclusion:**

Australia has had several successes towards UHC. However, access to health services poses significant challenges among specific priority populations and rural residents. To achieve universality and equity of health services, health system efforts (supply- and demand-side policies, programs and service interventions) are required to be implemented in multilevel health systems. Implementation of targeted health policy and program approaches are needed to provide comprehensive PHC and address the effects of structural disparities.

## 1. Introduction

Universal health coverage (UHC) is the global commitment to achieving quality health services for all, comprising a range of essential health services at the population level without financial hardship [1]. The UHC service coverage index (SCI) measures broader categories of services (maternal and child health services, infectious and noncommunicable diseases (NCDs), and service capacity) and access [2]. The population-level coverage is the uptake of services among socially, economically, and geographically defined groups. Financial coverage includes risk protection from financial hardship and measures out-of-pocket (OOP) expenditure while accessing health services [3]. Tracking the SCI is crucial to identifying the performance of health systems towards UHC, including universality and equity of health services and health outcomes.

Australia is one of the countries with a high UHC SCI (89%) [4]. In 2021, Australia's overall health system performance was the third among eleven high-income countries (HICs) [5]. Such high health system performance is possible through its Medicare program, a universal public health insurance scheme favouring values of universality and equity [6]. The provision of Medicare allows all Australian citizens and permanent residents to access health services at little or no cost [7], providing free inpatient care and medication subsidies as defined in the Medical Benefits Schedule and Pharmaceuticals Benefit Schedule [8]. The federal policy also supports private health insurance (PHI) initiatives by ensuring greater access to hospital care and allied health services through PHIs that are not included in the Medicare program (e.g., dental and physiotherapy services) [9, 10].

Australia has a mixed (public and private) health care delivery system. The federally funded initiatives for PHC include general practitioners (GPs), Aboriginal Community-Controlled Health Organisations (ACCHOs), and primary health networks (PHNs). Most primary care services are provided by GPs (public and private), primarily located in inner urban areas [6]. Health organisations such as ACCHOs provide comprehensive PHC services to Indigenous communities [11, 12], while PHNs commission health services and service gaps [13]. In addition, each state has its health department, comprising regional bodies called local health networks (local hospital networks, local health districts, health service regions, health organisations, or hospitals and health services) [13]. At the local level, local councils also run community health clinics which provide several preventive health services (e.g., childhood immunisation and antenatal care) [13].

Despite Australia's universal health care system, disparities and challenges remain with access to health care, affordability of health services, and care processes among certain disadvantaged groups and remote and rural areas, for example, people of Aboriginal and Torres Strait Islander backgrounds (respectfully referred to as Indigenous population hereafter), culturally and linguistically diverse (CALD) populations (those who were overseas-born and who speak languages other than English at home) [14], sexual minorities (lesbian, gay, bisexual, transgender, and intersex) [15], and residents of remote and regional areas. Indigenous Australians comprise 3.3% of the total population [16]. Compared to non-Indigenous and urban areas, health indicators and access to health services are poor among disadvantaged groups. Compared to non-Indigenous people, Indigenous Australians have eight years shorter life expectancy at birth [17], while 12 years shorter for Indigenous Australians living in remote areas [18]. Indigenous Australians (of remote areas) have experienced a high burden of infectious (burden of skin, eye, and respiratory infections) and NCDs such as mental and substance use disorders, and obesity [19, 20]. One in four (26%) Australians belongs to the CALD groups, while nearly one in five (17.9%) is from non-English-speaking countries [21]. People of CALD backgrounds, such as refugees, suffer from mental disorders and obesity compared to the general population [6, 22, 23]. Even Australian-born immigrants and non-English-speaking migrants have a high burden of mental disorders and poor access to health services [24]. Furthermore, in residential aged care facilities (RACFs), elderly multicultural people comprise more than one in five (22%) [25]. Furthermore, many of these disadvantaged populations live in regional areas, have poor access to health services, and experience a high burden of diseases but have limited delivery of and access to health services [26–28]. Sexual minorities also experience stigma and sexual identity-based discrimination while accessing health services at the point of care [29, 30]. Moreover, these disadvantaged populations have faced high care costs, lack of timely care, and care process (preventive, safe, coordinated care, and engagement and patient preferences) [5]. These indicate persistent inequity in health outcomes in the context of Australian universal health care system. Health inequities are contributed by social determinants of health such as disparities in socioeconomic status and income and discrimination at individual, organisation, and system levels [31].

Evidence suggests that there have been barriers to access to health services and inequities in health outcomes among priority populations (such as Indigenous, multicultural populations, sexual minorities, and residents of rural and remote areas) compared to non-Indigenous Australians. However, there is a dearth of systematic synthesis of the evidence explaining the successes and challenges addressing the health needs of these populations and rural residents and what drivers influence (positively and negatively) while accessing health services. Thus, this study synthesized successes and challenges towards health care for all, and their multilevel drivers (operating within the health system and beyond) influencing in access to and utilisatioin of health services . The findings of this study could inform policy towards universality and equity of quality health services in the context of the universal health system in Australia.

## 2. Methods

### 2.1. Study Design

We conducted a scoping review of the literature using a scoping review framework proposed by Arksey and O'Malley, which involves six steps: identifying the research question; identifying relevant studies; selecting studies; charting data; collating, summarizing, and reporting results; and consultation (optional) [32]. A scoping review helps synthesize and analyse existing research on a particular topic to map out the breadth and depth of the available evidence. We followed some components of Preferred Reporting Items for Systematic Reviews and Meta-Analyses extension for scoping review (PRISMA-ScR) protocol to report the findings of this review study [33] (Supplementary information, Table S1).

### 2.2. Identifying the Research Question

We identified the research question focusing on the successes, challenges, and drivers of the Australian health system towards UHC. We conceptualised four main concepts: health systems/services, successes/challenges, UHC, and Australia. These concepts helped to define search strategies. We assumed that the proposed research question is broad to provide a breadth of issues to be explored in the review. The research question was further clarified by preliminary discussion among authors, who agreed on the scope, breadth, and significance of the topic and therefore decided to proceed with the review.

### 2.3. Identifying Relevant Studies

Three databases were searched: PubMed, Embase, and Scopus. Search terms were identified and organised under four domains (supporting information, Table S2): UHC, health services/systems, successes and challenges, and Australia (states/territories). We included quantitative, qualitative, and mixed method studies published from 1 January 2010 to 30 July 2021 and published in English. We excluded study protocols and letters to editors.

### 2.4. Selection of Studies for Review

**Figure 1:**
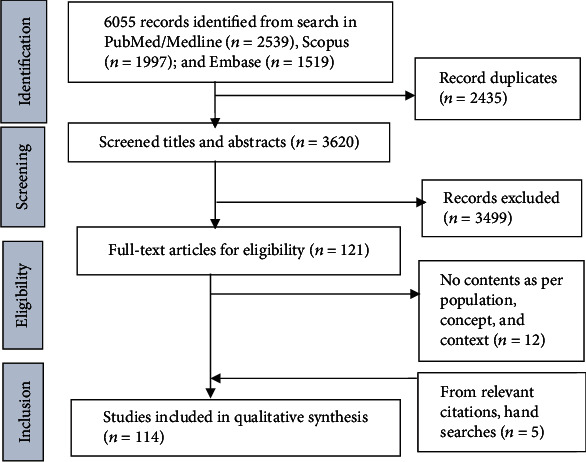
PRISMA-ScR flowchart showing the selection process of studies in the review.

The first author developed the search strategy, and the second author reviewed and verified it independently. Then, the first author searched records in databases and assessed the titles and abstracts of selected studies to evaluate their eligibility. Next, full-text studies were assessed and discussed with the second author. After consensus among the author team, studies were included in the review (Figure 1). We selected and included studies in the context of the purpose of our review, rather than based on the quality of the individual study included in the review [34–36]. To effectively answer the research question, we adopted the PCC framework recommended by the Joanna Briggs Institute that consists of the population (e.g., all population groups as services users and health workforces), concept (e.g., PHC services, primary care, and GP services), and context (Australian health system) [37]. We adopted the PRISMA-ScR as a reporting tool for the scoping review while the PCC framework guides the selection of relevant studies for the review [38–40].

### 2.5. Charting the Data

A data-charting form (excel sheet) was developed to extract data from each study covering author, year, country, type of study, key concepts, and main findings related to the research question (template, supplementary information, Table S3). A descriptive-analytical method was used to extract contextual or process-oriented information from each study.

### 2.6. Data Analysis and Synthesis

We analysed data using an inductive approach and generated themes. During thematic analysis, we followed procedures such as familiarising data, generating the main point from the data, searching for themes, reviewing themes, defining themes, and writing them narratively [41, 42]. First, the successes and challenges of a range of health services were described. Drivers of successes and challenges were described using a multilevel framework (comprised of proximal, intermediate, and distal level) [43]. Proximal level drivers/factors include those that directly operate and influence the users and providers at the service delivery level. Intermediate level drivers/factors act at a higher than the proximal level (higher than the service delivery level), which operates at the system and community level and facilitates service delivery and utilisation. Distal level drivers/factors operate at the policy level and influence the structural factors.

## 3. Results

A total of 114 studies were included in this review (Figure 1). This review identified several successes and challenges of accessing and utilising health services and their underlying drivers towards UHC in Australia.

### 3.1. Successes

There were many successes in accessing and utilising health services for infectious disease, maternal and child health (MCH), and NCDs. Studies reported the increased utilisation of HIV testing, preexposure prophylaxis (PrEP) and sexually transmitted infection (STI) services to vulnerable people, CALD groups (e.g., migrants), reduced gaps in STI services, and vaccination coverage for hepatitis, including those who are ineligible for Medicare [31, 44–53]. Additionally, people's perceived need for health care increased the use of condoms, increased the uptake of maternity and abortion services, and improved postnatal contraception among Indigenous women [54–64]. The CALD groups have improved the choice and control of contraceptive and care provision for MCH services [62, 63, 65–69] and immunisation and abortion services in rural areas [53, 55, 57, 70, 71]. Furthermore, communities were enabled to address the complex sexual health needs of migrants [48, 49, 66, 72].

Similarly, building trust, breaking cultural stereotypes, and rapid referrals for services helped improve the prevention and treatment of NCDs [15, 30, 69, 73]. Indigenous providers ensured culturally responsive care and encouraged Aboriginal people to tobacco cessation and screening and treatment of NCDs [27, 61, 63, 64, 74–81]. Furthermore, drivers such as staff engagement partnership with community people, gender matching with health staff, reducing cost and travel time, and telehealth approach improved care provision and utilisation of health services [57, 59, 60, 75, 82–85]. Using PHC services by Indigenous and remote people decreased risks, promoted preventive care, halted disease progression, and reduced hospitalisation rates and time for NCDs [51, 78, 86–90]. Better access to prescription medications reduced the risk of complicated diseases and saved hospitalisation costs in remote areas [13, 50, 51, 69, 89, 91–93].

### 3.2. Challenges

There were some challenges to UHC in Australia. For instance, access to PrEP services was constrained for people with chronic health conditions [15, 46, 47, 68, 91, 92, 94–99]. People with low income and living in rural areas had poor access to PrEP services [45, 46, 57, 100, 101]. Additionally, rural populations had poor access to immunisation and maternity (e.g., abortion) services [45, 48, 49, 51, 57, 100–106]. The CALD groups and remote residents faced unmet needs of postpartum contraceptives [54, 65, 73, 100, 107, 108]. Indigenous communities faced several barriers (e.g., language, transport facilities, and costs) that hindered the provision of childbirth and family planning (FP) services [51, 54, 105, 109]. Health programs for the CALD groups were short term, leading to discontinued care and low utilisation of services (e.g., FP and abortion) [49, 54, 57, 65, 100–102, 105, 107, 108, 110]. Furthermore, inadequate engagement of providers and users and staff shortages decreased maternity services to Indigenous populations [51, 63, 79, 105, 109]. Poor skills and capacity of the health workforce and difficulties in identifying problems impacted the promotion, surveillance, and delivery of MCH services in rural care health facilities (HFs) [46, 51, 73, 82, 86, 100, 101, 107, 111–120]. Moreover, there was a lack of funding for health programs for the CALD population, and a treatment-focused care model also constrained health services [49, 97, 116].

With NCD care and treatment among Indigenous women, there were challenges (uncomfortable and invasive procedures, negative experiences, anxiety, embarrassment, lack of privacy, low-risk perception, a lack of behaviour change activities, and education and misperceptions on NCD screening) which access to health service utilisation [54, 77, 84, 98, 121–124] that contributed to the decreased NCD-related health service utilisation (e.g., substance abuse, mental health, and screening of cancers) [27, 55, 61, 73, 103, 125, 126]. Additionally, there was increased premature onset and incidence of NCDs due to poor providers' understanding, social underpinnings of poor health, and a lack of social and emotional wellbeing [61, 77, 121]. The lack of after-hours services for NCDs in remote areas further compounded in achieving clinical risk reduction targets in high-risk clients for prevention and treatment [27, 112, 121, 123, 127, 128].

There were limited health resources for programs related to migrant health, resulting in a lack of programs and high unmet health service needs [48, 98, 102, 129]. Funding challenges compounded by the competing interests in resource allocation hindered care provision for chronic health conditions [15, 46, 87, 91, 92, 94–96, 112, 130]. High care costs in multimorbidity and lack of insurance increased the OOP expense [97, 98, 131–133]. Fragmentation of funding for health programs also influenced inadequate access to high-need groups, including cervical cancer screening and cessation of tobacco consumption [27, 78, 79, 83, 84, 96, 109, 126, 130, 133–136]. Moreover, people from CALD and sexual minority backgrounds experienced structural challenges (e.g., no full-time work and stereotyping discrimination) that further increased health inequities [15, 30, 66, 72, 102, 104, 137, 138].

### 3.3. Multilevel Drivers

Several drivers (enablers and barriers) of health services were identified (Table 1). These drivers are described under three levels: proximal (at a delivery point), intermediate (community and organisational level), and distal (structural or political level).

### 3.4. Proximal Level Drivers

Seven broader drivers were identified under the proximal level, including four demand-side drivers (health literacy, sociocultural factors, users' language, and reaching to HFs) and three supply-side drivers (providers) (e.g., providers' behaviours, quality of health workforce, and provision of services at HFs).

#### 3.4.1. Health Literacy

Health literacy on health needs, rights, and risk perception awareness increased health service utilisation. For instance, providing information and empowerment influenced the control of women's reproductive health rights, peer-led health promotion and support, and knowledge on the safe use of essential medicines for MCH services [54–58]. Moreover, improved social interaction and communication, self-awareness, and perceived benefit of smoking cessation enabled the health promotion of NCDs [59, 78, 90, 99, 119, 139] and the prevention of infectious diseases [28, 51]. Nevertheless, the low-risk perception of women has resulted in poor access to MCH services in rural Indigenous populations [45, 48, 49, 51, 57, 100–106]. Similarly, lack of confidence in perceived shame, familiarity with methods, inadequate information, and misperceptions decreased the use of postpartum contraceptives in rural areas [54, 65, 73, 100, 107, 108]. Some remote Indigenous populations' frustrations over the treatment and lack of trust in the providers contributed to the low utilisation of health services [51, 63, 64, 79, 84, 109, 140]. Finally, conflicting messages from health care providers, low-risk perception of diseases, and literacy gaps of migrants were reasons for poor health care utilisation [49, 66, 98, 102, 104].

#### 3.4.2. Sociocultural Factors

Migrants having skills to deal with two cultural identities contributed to better utilisation of health services [66, 69, 72, 103]. Informed choice and rights-based approaches, disclosure of the identity of sexual minorities, users' engagement in trust building, and breaking cultural stereotypes were crucial factors for preventing and treating NCDs [15, 30, 69, 73]. Poor capacity to overcome fears, shame, and negative experiences motivated screening NCDs and tobacco cessation among some Indigenous populations [74, 78–81]. In contrast, CALD populations (especially migrants) faced social isolation, lack of male involvement and support, loss of connection to migrants' original culture, and gender-related vulnerabilities in the new settlement that influenced low utilisation of health services [15, 30, 66, 72, 102, 104, 137, 138]. Additionally, inadequate discussion on treating STIs among migrants included stigma, cultural values, and norms associated with sexual relations [28, 45].

#### 3.4.3. Users' Language and Communication

Users' cultural sensitivity to overcome stereotypes improved the utilisation of health services [55–58, 62, 112, 119]. For example, informal talk with providers and peers was identified to mitigate language barriers and change the social norms and values on health and diseases [28, 90]. Additionally, providers of similar ethnic backgrounds (with users), cross-cultural workers, and gender matching with staff enhanced the uptake of information and understanding of cultural values and practices [59, 60, 74, 77, 79, 143]. However, failure to understand languages, a lack of fluency and confidence in speaking English (e.g., during phone bookings), and high expectations of migrants constrained in accessing PHC services [15, 30, 66, 72, 102, 104, 137, 138].

#### 3.4.4. Reaching to HFs

Adequate transportation facilities, maternity units in public hospitals, and affordable services at delivery points improved reaching HFs for services [54–58]. Early antenatal care visits and short travel times have increased the utilisation of MCH services [53, 55, 57, 70, 71]. Additionally, timely and early testing services decreased coverage gaps of STIs and hepatitis vaccination in remote areas [50–53]. Using PHC services by disadvantaged groups reduced the risk of complications, improved initial treatment for NCDs, and ultimately decreased hospitalisation rates [51, 78, 86–90]. However, financial problems such as expensive drugs, billing, and payment issues without Medicare increased OOP expenditure of PrEP services for people with chronic conditions [15, 46, 47, 68, 91, 92, 94–99]. Furthermore, high care costs hindered access to PrEP services for people in low-income and rural areas [45, 46, 57, 100, 101]. Limited access to transportation services, long travel time, lack of accommodation, and high mobility of communities resulted in MCH services in remote Indigenous populations [45, 48, 49, 51, 54, 57, 100–106, 109].

#### 3.4.5. Providers' Behaviours and Communication

The provider's role in overcoming stigma and shame improved trust and encouraged Aboriginal populations in the screening of NCDs and tobacco cessation [74, 78–81]. Understanding participants' language and interpreters enabled providers to address the complex sexual health needs of migrants [48, 49, 66, 72]. Moreover, using culturally sensitive language and ensuring confidentiality improved the care provision and utilisation [57, 59, 60, 75, 82–85]. Providers' capacity to maintain confidentiality and privacy and minimise shame increased utilisation of PHC services [59, 60, 74, 77, 79, 143]. Providers' clinical accountability, willingness to self-educate, and nonjudgemental and inclusive language effectively delivered services for sexual minorities [30, 144]. Increased clients' trust and providers' analytical capacity enhanced quality service delivery for underserved communities [48, 71, 117]. Nevertheless, providers' language barriers and lack of cultural competency hindered FP and MCH services [51, 54, 105, 109]. There were inadequate providers' social interaction skills (cultural competency and confidentiality and social isolation) while providing services to the CALD groups, resulting in inadequate utilisation of MCH services [49, 54, 57, 65, 100–102, 105, 107, 108, 110].

#### 3.4.6. Quantity and Quality of Workforces

Dedicated clinical time by a multidisciplinary team has increased health service utilisation [44–47]. Strengthening the local health workforce and cultural sensitivity to overcome stereotypes improved utilisation of services [55–58, 62, 112, 119]. Providers' trusted relationships, responsiveness, and shared cultural backgrounds addressed community needs in urban Indigenous health [72, 90, 125, 143]. Expanded roles of Indigenous providers ensured culturally responsive care for preventing and treating NCDs [27, 61, 63, 64, 74–77]. The involvement of young GPs in RACFs and the provision of multidisciplinary (social workers) teams increased the uptake of promotion, prevention, and treatment for NCDs [59, 98, 101, 103, 119, 139, 143]. Nevertheless, high staff turnover, inadequate technical skills and experiences, and lack of follow-up reminders affected the prevention and treatment of infectious diseases, especially in rural areas [45, 46, 51, 107, 117]. Shortage of health workforce and lack of clarity on provider roles decreased the utilisation of MCH services among Indigenous groups [51, 63, 79, 105, 109]. Additionally, providers' poor understanding of participants' language and respect for migrants' beliefs, lack of interpreters' services, lack of reminder calls, and insufficient training of GPs influenced care provision of MCH services to migrants [48, 49, 67, 68, 102, 104, 116, 138]. Providers' inadequate understanding of social underpinnings and well-being increased premature onset and incidence of NCDs [61, 77, 121]. Competing clinical priorities, limited roles of the PHC workers, and the capacity of NCD care influenced the prevention and control of NCDs (e.g., substance abuse and cancer screening) [27, 61, 126].

#### 3.4.7. Provision of Services at HFs

Culturally safe and continuity of midwifery care and an increased role of nurses in sex education improved choice and control of contraceptives and care provision for MCH services [62, 63, 65–69]. Provision of timely and family-centred care at a reduced cost and flexible hour services closer to home or outreach settings increased health service utilisation [53, 55, 57, 70, 71]. Welcoming and nonjudgemental attitudes of providers fostered culturally appropriate services to people with the greatest need in rural areas [53, 55, 88, 101, 105]. Follow-up and reminder services have improved health services for HIV patients [28, 51]. Routine care provided by the preferred gender of providers and provided in prearranged group appointments improved testing and treatment of HIV and STIs among migrants [47–49]. However, the shortage of workforce, closure of rural maternity units, and lack of rapid referral services negatively influenced MCH services in rural HFs [51, 82, 100, 101, 107, 111–117]. Providers' inadequate competency and unavailability of after-hours services for NCDs challenged the achievement of clinical targets of risk reduction and treatment in high-risk clients in remote areas [27, 112, 121, 123, 127, 128]. Lack of care coordination and poor understanding of the practical realities made it challenging to address rural areas' complex/changing care needs [27, 63, 84, 96]. Minimal attention to health promotion interventions increased the burden of diseases and poor service delivery NCDs [58, 125, 128, 135, 136].

### 3.5. Intermediate Drivers

Some community (such as engagement and participation) and organisational (models of care, financial resources, health workforce, and evidence use and monitoring) drivers were reported to influence access to and delivery of health services in Australia.

#### 3.5.1. Community Engagement and Participation

Community interaction (interpersonal and intrapersonal) with local and Aboriginal populations improved the perceived need and encouraged postnatal contraception [54, 59–64]. Local community engagement and women's empowerment in health rights increased the demand for health services in rural areas [28, 90]. The embedded relationship of providers with Aboriginal people, partnerships, coordination, and engagement with the government enhanced access to services among underserved communities [27, 61, 63, 64, 74–77]. Additionally, partnerships for collaboration within the Medicare Locals model and community-level work were adequate for the design and services for people from sexual minorities [15, 30, 137, 144]. However, poor community engagement of target groups, limited health resources for refugees, and lack of resources and organisation at HFs led to high unmet needs and poor access to health care [48, 98, 102, 129]. Poor communication and insufficient understanding of contexts undermined health promotion and preventive programs [111, 112, 130]. Lack of effective collaboration with users and disjointed and fraught providers' relationships contributed to the utilisation and provision of health services [49, 54, 57, 65, 100–102, 105, 107, 108, 110].

#### 3.5.2. Programs and Models of Care

Some examples of health programs that support the delivery of health services included Aboriginal Immunisation Healthcare Worker, Elmore Primary Health Service, Aboriginal Family Birthing Program, Southgate Model of CPHC program, and the Central Australian Aboriginal Community-Controlled Health Service model [53, 81, 85, 88, 105, 133, 145–147]. Midwifery-led continuity of care models shifted the power dynamic from a hierarchical system that increased the uptake of MCH services in Indigenous populations [62, 80, 105, 110]. Stronger regional collaborations of local organisations and involvement of local governments improved care coordination and health system governance [13, 69, 85, 90, 96, 113, 119, 148, 149]. Clinical networks, team-based primary care, and care coordination framework enabled system-wide changes [86, 96, 148–152]. Service coordination and quality improvement initiatives have facilitated the sustainability of services in rural PHC settings [70, 71, 118, 146]. Nevertheless, mainstream PHC programs were short term and lacked focused interventions that resulted in discontinued care and had challenges in their evaluation [49, 67, 102, 104, 116]. The lack of alternative models of care, overcrowding, and nonfunctioning health infrastructure influenced poor service delivery for NCDs [58, 125, 128, 135, 136]. The current health system lacks clinicians or a choice of practitioners to address the needs of sexual minorities [15, 30, 137]. Unclear roles and responsibilities overlap between public health and primary care, and a poor focus on frontline health services influenced the commissioning of health services under PHNs [55, 116, 149]. With a lack of a national policy on catch-up vaccination, a medically centred model of care also constrained MCH services for migrants [49, 97, 116]. The health system efficiency was hindered due to ambiguities in the federal/state divided responsibilities for PHC, medico centricity and privatisation, and highly bureaucratic but unclear accountability mechanisms [13, 96, 113, 115, 150].

#### 3.5.3. Financial Resources

Targeted copayment, bulk billing, primary care incentives, and commissioning services mitigated some financial constraints of health care [13, 69, 73, 91–93, 118]. Furthermore, blended payment methods, monitoring of OOP expenditure, and data-informed decision-making in program regulation and performance were effective planning and service delivery [69, 84, 132, 148, 151, 153]. Moreover, factors of better access to prescription medications that increased utilisation of PHC services were flexibility and regional funding for Indigenous people, Mandatory Integrated Public and Private Health Insurance incentives, reduction in copayment cap, and saved hospitalisation costs in remote areas [13, 50, 51, 69, 89, 91–93]. However, lack of insurance also hindered the care for CALD populations with multimorbidity, resulting in high OOP expenditure [97, 98, 131–133]. Insufficient funding, competing priorities in resource allocations, and a lack of national programs on chronic conditions have resulted in inadequate access to high-need groups [79, 109, 130, 136]. In addition, there was fragmentation of funding that hindering the design and implementation of NCD-related health programs (e.g., cervical cancer screening) among certain disadvantaged groups (e.g., migrants) [27, 78, 83, 84, 96, 126, 133–135].

#### 3.5.4. Health Workforce Management

Partnership with universities for Indigenous midwives and Aboriginal people strengthened the inclusion and addressed the shortage of culturally competent workforce [57, 59, 60, 75, 82–85]. Increased regional maternity care workers and Indigenous midwives contributed to improving health service delivery [62, 80, 105, 110]. Quality supervision, supervised GP training, professional development programs, learning opportunities, interprofessional education, and management systems attracted and enhanced the retention of workforces [48, 71, 117]. Nevertheless, there were challenges in health workforce management, such as a lack of funding for nurse innovation, insufficient training, and capacity-building opportunities [51, 82, 100, 101, 107, 111–117]. Additionally, studies reported high workloads, administrative burden, shortages and high turnover, inequitable distribution, opportunity costs of GP services in RACFs, poor trust and burnout, and lack of skills and experiences [27, 73, 82, 111–116, 133, 148]. Furthermore, other challenges of health workforce governance included poor understanding of administrative and management functions and inadequate skill experience [13, 96, 113, 148, 154].

#### 3.5.5. Evidence Use in Planning and Monitoring

Standard setting and benchmarking, tailoring using comparable data, monitoring, and surveillance improved the program performance by increasing the inclusivity for PHC services [15, 60, 70, 87, 98, 106, 107, 120, 127, 144]. Timely collection of consistent data from multiple sources and collating and creating visual indicators strengthened program management [15, 55, 85, 107, 120, 137, 144]. Adopting board training, accreditation, and national quality and performance framework improved quality-of-care governance [86, 118, 119, 148, 153]. Using codesign principles, tailored community-driven and bottom-up health planning in Indigenous communities was reported to incorporate local circumstances integrated care for multimorbidity and chronic diseases [61, 62, 76, 85, 136, 143]. Surveillance systems and timely reporting strengthened the screening of NCDs in rural areas [70, 87, 106, 120, 127]. The use of records of care and quality indicators provided benchmarking of PHC services [15, 30, 86–89, 144]. On the other hand, lack of quality data and poor understanding of sensitivity in surveillance hindered the PrEP services [46, 73, 120]. Lack of comparable information affected the planning of care prevention of STI treatment in rural HFs [51, 107, 117]. Inadequate data on quality of care, missing link of translation of evidence into practice, and inadequacy to identify problems impacted the promotion, surveillance, and delivery of MCH services [46, 73, 86, 114, 118–120]. Poor coordination of health planning, inadequate funding of PHNs, and performance and regulatory requirements resulted in poor multilevel governance [13, 96, 113, 148, 154].

### 3.6. Distal Level Drivers

#### 3.6.1. Socioeconomic and Ethnic Diversities

At the higher sociopolitical level, there were drivers of UHC in Australia, such as the Medicare program. The Medicare (universal public health financing program) is one of the distal factors of success for the UHC [6]. However, some structural and socioeconomic challenges hindered health service delivery and utilisation. For instance, lack of full-time work, economic hardship, and experiences of stigma and discrimination in employment reinforcing existing exclusion increased health inequities, especially among the CALD groups [15, 30, 66, 72, 102, 104, 137, 138, 142]. Moreover, systematic discrimination and dominant culture at HFs limitedly captured the historical/cultural dimensions of Indigenous values and culture; Indigenous knowledge was undervalued, threatening the long-term viability of sustainable Indigenous programs [63, 64, 79, 84, 96, 109, 130, 140].

## 4. Discussion

This review identified several successes and challenges and their drivers towards UHC in Australia. Key achievements included increased utilisation of infectious disease services, decreased equity gaps, improved perceived needs of MCH services, and preventive care for NCDs. Nevertheless, there were challenges, such as poor access to health services for priority populations. These groups had poor access to services for multimorbidity, high unmet health needs, and poor achievement of clinical targets of risk reduction and treatment of NCDs. Driving factors related to users included health literacy, sociobehavioural, users' language and communication, and reaching HFs; provider-related drivers were behaviours, quantity and competency of health workforces, and provision of health services. At the community and health facility level, drivers were community engagement, health policy and programs, financial resources, evidence-based planning and monitoring, and mobilisation of the health workforce. Finally, distal level drivers were the lack of inclusion of all taxpayers in the Medicare program and socioeconomic disparities.

### 4.1. Universal but Not for All

Despite the high UHC SCI (89%), certain groups in Australia (e.g., Indigenous and CALD groups) have poor access to essential health services and have equity gaps in terms of access to health services, status, and health outcomes. These disadvantaged groups and residents living in rural areas had poor access to health services and health status [155]. The burden of NCDs and chronic diseases was high among the Indigenous groups [19], and remote residents suffered from chronic infections (e.g., skin, eye, and respiratory) [20]. People from CALD backgrounds have a high risk of infectious diseases in the early years of migration, while there are increased NCDs as the length of stay lasts longer and among first-generation migrants [156, 157]. Refugees suffered from mental disorders (highest among Tamil immigrants) than general populations [22, 23]. Among migrants of first- and second-generation migrants, there was a higher burden of mental health status among non-English-speaking migrants, and they had poorer access to services than Australian-born immigrants [24].

Additionally, people from the CALD groups encountered multiple challenges at HFs (by providers) and resettlement (communities) [158, 159]. While some taxpayers (e.g., temporary migrants) lack access to Medicare benefits while accessing health services and need mandatory purchase of PHIs, they also struggle to renew premiums [10, 160]. People from sexual minority backgrounds experienced stigma and discrimination while accessing health services [29]. Rural residents experience an increased burden of infectious diseases and NCDs, and rurality further exacerbates the difficulties in reaching HFs/providers for health services [26–28, 59, 66, 103]. Additionally, rural residents face problems with timely access to GP services in rural areas and have high bulk-billing rates [70, 87]. People already left behind should be prioritised with targeted policies and program approaches considering the multilevel health system contexts.

### 4.2. Breaking Barriers through Multilevel Approaches

Addressing inequities in health services among disadvantaged populations requires both demand- and supply-side interventions at the individual level (service user and provider level), intermediary level (community and health facility level), and system level (broader system level). Individual-level strategies can potentially address the social determinants of service users (lifestyle, behavioural, and health-seeking factors) and drivers of health care providers (skills, attitude, and competency). Intermediary strategies address the issues of community and health organisational level, while system-level strategies could address the structural challenges.

### 4.3. Engaging and Empowering Disadvantaged Populations

User-level factors such as health literacy, sociobehavioural factors, language, and access to HFs can be addressed by demand-side approaches. For instance, cultural norms, family interpersonal relations, and values influence health literacy and the need to address the gaps in health care [161]. Additionally, interactive learning, such as group discussions, peer-support, and digital tools, helps service users take the initiative and ownership and increase the utilisation of health services [162, 163].

People can have sociopsychological barriers (e.g., cultural differences, perceived shame, stigma during health care-seeking, and poor confidence in communication) that can be addressed by improving users' and providers' communication skills. Perceived cultural stigma and shame, poor patient-provider interactions, and treatment adherence lead to poor outcomes [164]. Creating common in group identities, promoting contact among perceivers, and enhancing social support and adaptive coping mechanisms can improve resilience to societal stigma and racial and ethnic stereotyping and build confidence in the system [165]. Furthermore, including intersecting factors of gender/cultural norms of the destination country in the migration process could mitigate cross-cultural trauma, isolation, and social disadvantages of migrants [166, 167]. Additionally, nonverbal communication (posters), mobile translation technology, and web-based interpretation services were found to address language barriers in health services [168]. Culturally appropriate communication interventions improved the health literacy of non-English-speaking migrants [169]. Telehealth interventions can mitigate financial and geographical barriers in remote and underserved communities. For instance, technology-based solutions such as phone or video consultations can minimise costs while accessing health services by providing services in flexible hours in rural areas [166].

### 4.4. Enhancing Health Workforce Capacity

Provider-related proximal level factors (e.g., providers' behaviour, quality, and competency) can be addressed by several supply-side approaches at the point of care, potentially improving the provision and delivery of health services. For instance, providing short courses on languages to providers and employment of diverse workforces can mitigate providers' communication, including cultural and linguistic obstacles between patients and providers [168]. Evidence suggests culturally responsive communication of providers improved access to PHC services among migrants in HICs [158]. Communicating in users' language and integrating interpretation services in communities with limited English, recording patients' language, and professional medical interpreters mitigated the providers' linguistic and cultural barriers at the provider's level [170–173]. A multidisciplinary team, multicultural and Indigenous providers, provision of interpreters, gender matching with users, and cross-cultural training could negate ethnic bias, stigma, and stereotypes and provide culturally appropriate care [43, 174]. At the point of care, cross-cultural communication through a diverse workforce can address language and sociocultural barriers [175, 176]. For example, Indigenous interpreters were a vital bridge between patients and providers in Canada [177]. After-hours services and reminder systems can also potentially improve the utilisation of primary health services. Tele reminder systems (e.g., short messages) were found to be effective in different ways (increasing the attendance of appointments, shaping behaviour across the spectrum of health care, and promoting healthy behaviours) [178]. The timely utilisation of PHC services and referrals can prevent excess hospitalisation rates and adverse health outcomes [179].

#### 4.4.1. Community Engagement and Partnership

Demand-side factors at the intermediary level (e.g., community engagement and participation) could increase the demand for health services. For instance, community-driven approaches to social mobilisation, inclusion in community health governance, and partnership with community organisations could improve awareness, empowerment, and participation, making people access health services [180]. Furthermore, such approaches strengthen the accountability of health organisations and providers. Studies revealed that community involvement and engagement in health care need confidence in the health systems and services [181, 182]. Improving communication and continuity of care and confidence in the system is imperative, significantly improving access to address the health disparities among disadvantaged groups [182, 183]. Australia's ACCHS model improved access to culturally responsive services through intersectoral collaboration, prevention, and promotive services [7, 184, 185]. Culturally competent care from providers and increased health literacy of priority populations have potential to improve health service utilisation [169].

#### 4.4.2. Organisational Responsiveness

Several strategies (related to health finance, health workforce, and health programs) can be employed to improve the organisational responsiveness to the delivery of health services. Firstly, health care financing interventions can reduce the equity gaps in health services. So health care financing policies require to move from a single-disease framework to multimorbidity, particularly for the elderly and low-income groups [186]. Universal public health insurance integrating individuals' income-based insurance premiums can address the OOP expenditure in disadvantaged populations [187]. Pooling public resources is vital to reducing financial risk, while private insurance can supplement public insurance initiatives [188]. Secondly, health workforce production, recruitment, and deployment are crucial for service delivery. Production and deployment strategies such as providing scholarships, loan repayment programs, increased salary, and professional support can mitigate staff shortages and frequent turnover [43]. Partnerships with the university for the Indigenous workforce, including targeted enrolment into training and higher education programs, addressing the broader health system to ensure a safe and supportive work environment and providing individual and family support could increase retention in rural and remote areas [189]. Preferential selection of rural students and distributed training in rural areas are associated with increased rural retention of health professionals [190]. Health system actors were required to be initiative-taking to design gender-sensitive, tailored, and contextual plans to meet the clinical targets in the high-risk groups. Thirdly, measurement standards and benchmarking can be helpful for the contextualisation of policies and for addressing local health needs [179]. Fourthly, previous studies revealed that local and Indigenous knowledge, partnership and collaboration with the local community, and shared decision-making were crucial for culturally responsive, continuity of care, and confidence with the health services [182–184, 191]. Finally, providing air transport services to bring patients to hospitals or bringing care providers to patients and integrating telehealth approaches also increased access to specialised care and reduced travel and waiting times by connecting patients from remote areas in HICs [192]. For example, hospitals and ambulatory care facilities provide health services in remote and underserved communities [43].

#### 4.4.3. Medicare Benefits for All

Australia's Medicare program is one of the best successes in universal access to health services. However, this program has several issues leading to inequities in health care. For instance, some taxpayers are excluded based on their visa status, high copayment gaps in remote and regional areas, and inadequate financial coverage for people living with multimorbidity and chronic conditions. Addressing these issues requires high-level political commitment and policy decisions. Additionally, CALD populations have intersectional (dis)advantages with multiple challenges: legal (e.g., asylum status), financial (expensive health care), cultural (discrimination), and geographical (distance to HFs) [182]. These structural factors are embodied in structural roots and influenced by factors of higher-level systems, which require long-term socioeconomic and political interventions. Moreover, including disadvantaged groups in the governance system, academic health programs and language and cultural courses in tertiary-level health curricula and programs could mitigate those structural challenges [43, 168]. Reducing the socioeconomic equity gaps and providing employment and work opportunities for marginalised people can address the upstream social determinants of health.

### 4.5. Implication for the Policy and Programs

Despite providing a universal health care system in Australia, some populations (e.g., Indigenous, CALD groups, sexual minorities, and remote and rural residents) have several challenges accessing and utilising health services. Some policies and programs for disadvantaged populations include Indigenous, CALD groups, and rural areas. However, evidence from this study revealed several challenges and their driving factors that need to be addressed for improved health services among priority groups. The findings of this study can have implications for the revisions of existing policies and programs for improved health service delivery and utilisation of services. There is a need for several supply-side and demand-side program interventions needed at different levels of health systems and communities. Particularly, the CALD groups have diversity within cultural, linguistical, socioeconomic, and geographical, and some populations within the diverse groups can have relative privilege in access to health services. In contrast, some might have suffered from multiple forms of intersectional disadvantages. Further studies are warranted to identify the most disadvantaged CALD groups and review the existing health program and policies.

### 4.6. Strengths and Limitations of the Study

There are some limitations of this study. Firstly, we conducted a narrative literature review and synthesized the findings of quantitative, qualitative, and mixed method studies. Therefore, meta-analysis was not considered appropriate because of the wide variability of studies with research design, study population, types of interventions, and outcomes. Like systematic review and meta-analysis, we could not describe in detail any variable that mediates health outcomes. Instead, as per the PRISMA-ScR protocol, we included all types of studies and synthesized the drivers of PHC services. This study has not adopted a strict quality assessment of the individual study included in the review. However, we adopted the scoping review protocol and PCC framework to guide our analysis [33, 193]. The current review is the qualitative synthesis of the range of studies (e.g., quantitative, qualitative, mixed methods, and policy analysis). This study has provided insights into the delivery and utilisation of PHC services in Australia. Future studies can further explore in-detail specific issues highlighted in this study.

## 5. Conclusion

Australia has made significant progress towards UHC and has several signatory successes in the health system, service delivery, and utilisation. However, Indigenous and CALD groups, sexual minorities, and rural residents face significant challenges in accessing and poorly utilising health services. The health system should employ multilevel strategies at the proximal, organisational, and system levels. Remarkably, the challenges at the proximal level can be addressed by using both supply- and demand-side approaches at the delivery points. Health program interventions need to focus on attaining UHC among those populations who are already left behind. Achieving UHC is vital to aspire for a targeted universalism focusing on universal access among disadvantaged groups and remote areas. Community engagement and strengthening the health system can enhance the delivery and utilisation of health services. Persistent inequities in accessing health services among and between ethnic and socioeconomic groups need to be addressed through targeted policies and programs.

## Figures and Tables

**Table 1:** Multilevel drivers influencing health care for all in Australia.

	Enablers	Barriers	References
Proximal level			
Health literacy (demand)	Awareness in risk perception, trust, information and empowerment, peer-led health promotion, social interaction, self-awareness, and informing perceived benefit.	Low-risk perception of disease, perceived shame, little confidence, lack of familiarity, inadequate information, misperceptions, poor trust, conflicting messages, literacy gaps, and lack of prior medical history.	[28, 45, 48, 49, 51, 54–59, 63–66, 73, 78, 79, 84, 90, 98–109, 119, 139–141].
Sociocultural factors (D)	Adaption with two cultural identities, informed choice, rights-based approaches, identity disclosure, users' engagement, rust-building, and breaking cultural stereotypes.	Lack of cultural safety, confidentiality, stigma, stereotypes, social isolation, lack of male involvement, lost connection to culture, inadequate discussion on STIs, sociocultural and religious influence, and social isolation of people with HIV.	[15, 28, 30, 45, 66, 69, 72–74, 78–81, 102–104, 137, 138]
Language and communication (D)	Cultural sensitivity to overcome stereotypes, informal talk, homogenous backgrounds, cross-cultural workers, and understanding of culture.	Failure to understand language, lack of fluency and confidence, and high expectations of migrants.	[15, 28, 30, 55–58, 62, 66, 72, 90, 102, 104, 112, 119, 137, 138, 142].
Reaching to HFs (D)	Distance to HFs, availability of transportation facilities, maternity units in hospitals, affordable services, early health care visits in pregnancy, short travel time, timely and early testing at point of care.	High OOP expenditure and costly drugs, financial constraints, billing, payment issues without Medicare, high direct and indirect costs, poor access to transportation, long travel time, high care costs, lack of accommodation, high transport mobility.	[15, 45–49, 51, 53–58, 68, 70, 71, 78, 86–92, 94–106, 109, 142].
Provider's behaviours and communication (S)	Overcoming fears/shame/negative experiences, improved trust, engagement, and coordination, expanded roles of Indigenous providers, understanding participants' language, use of interpreters, use of culturally sensitive language, ensuring privacy and confidentiality, cultural representation, minimise shame, willingness to self-educate for minorities, practitioners' nonjudgemental approach and inclusive language, trust, knowledge to reduce stigma.	Language barriers, lack of culturally appropriate skills, inadequate social skills in exchanging information, lack of confidentiality, insufficient cultural competency, cultural disengagement, and social isolation.	[15, 30, 48, 49, 51, 54, 57, 59, 60, 65, 66, 71, 72, 74, 75, 77–85, 100–102, 105, 107–110, 117, 137, 143, 144]
Quantity and quality of workforce (S)	Dedicated clinical time, multidisciplinary team, local workforce, cultural sensitivity to overcome stereotypes, trusted relationships, responsiveness and shared cultural backgrounds, Indigenous providers, access to information to non-Indigenous providers, young GPs, the inclusion of psychologists and social workers, bilingual community educators.	High staff turnover, lack of capacity-building opportunities, inadequate technical skills and experiences, follow-up reminders, clinical complexity, shortage of Indigenous workers, inadequate engagement of providers with users, lack of clarity on provider roles, lack of interpreters or inconsistent services, lack of reminder calls, lack of respect of migrants' beliefs and cultural safety, insufficient training, providers' inadequate understanding, competing for clinical priorities, limited roles of PHC workers on NCDs.	[27, 44–49, 51, 55–59, 61–64, 67, 68, 72, 74–77, 79, 90, 98, 101–105, 107, 109, 112, 116, 117, 119, 121, 125, 126, 138, 139, 142, 143]
Provision of services at HFs (S)	Culturally safe and continuity of midwifery care, timely family-centred service package care, reduced cost in flexible hours, package at HFs, closer to home or outreach, and home visits, welcoming and nonjudgemental attitudes of providers, follow-up service recalls to HIV patients, reminder systems, frequent care, services provided by the preferred gender, prearranged group appointments, community radio, ethnic newspapers and posters in the dissemination of pretravel health information.	Closure of rural maternity units/shifts, unavailability of and difficulties in operating after-hours services, lack of rapid referral, insufficient training and capacity-building opportunities, inadequate knowledge and experience, logistical barriers, lack of care coordination, poor understanding of the practical realities, minimal attention on health promotion interventions.	[27, 28, 47–49, 51, 53, 55, 57, 58, 62, 63, 65–71, 82, 84, 88, 96, 100, 101, 105, 107, 111–117, 121, 123, 125, 127, 128, 135, 136, 142]

Intermediate level			
Community engagement and participation (D)	Community interaction with local and Aboriginal people, interpersonal communication with providers, contextual adaptation, community engagement, community representation, women empowerment, embedded relation of providers, partnerships and engagement with the governments, partnerships for collaboration within the Medicare Locals model and community-level work, use of bilingual community educators.	Lack of targeted community involvement, limited health resources for refugees, lack of resources and logistics, poor communication and information, insufficient understanding of contexts, lack of effective partnerships and collaboration with users, and disjointed and fraught providers' relationships.	[15, 27, 28, 30, 48, 49, 54, 57, 59–65, 74–77, 90, 98, 100–102, 105, 107, 108, 110–112, 129, 130, 137, 142, 144]
Programs and models of care (S)	Community-controlled health services, midwifery-led continuity of care, strong regional collaborations between PHNs and local organisations, understanding the difference between primary care and public health, involvement of local governments, service coordination and quality improvement initiatives, clinical networks and planned terms of references, team-based primary care, and care coordination framework.	Discontinuity and short-term programs, challenges in program evaluation in mainstream PHC programs, lack of alternative models of care, nonfunctioning health hardware, health system lacks of clinician or practice choices to address the needs of sexual minorities, unclear roles and responsibilities overlap between public health and primary care, lack of a national policy on catch-up vaccination, medically centred model of care, ambiguities in the federal/state divided responsibilities for PHC, medico centricity and privatisation, unclear accountability mechanisms, and high bureaucracy.	[13, 15, 30, 49, 53, 55, 58, 62, 67, 69–71, 80, 81, 85, 86, 88, 90, 96, 97, 102, 104, 105, 110, 113, 115, 116, 118, 119, 125, 128, 133, 135–137, 145–152]
Financial resources (S)	Targeted copayment, bulk billing, primary care incentives and commissioning of services, blended payment methods, single fundholding arrangements, and monitoring OOP, technology effectively generated data for informed intelligence and decision-making, flexibility in regional and funding for Indigenous people, Mandatory Integrated Public and Private Health Insurance incentives, reduction in copayment cap, better access to prescription medications.	High care costs in multimorbidity (lack of insurance), insufficient funding, competing priorities in resource allocations, a lack of national programs on chronic conditions, fragmentation of funding and services, lack of financial incentives, had no support for high taxpayer priority access.	[13, 27, 47, 50, 51, 68, 69, 73, 78, 79, 83, 84, 89, 91–93, 96–99, 109, 118, 126, 130–136, 148, 151, 153]
Health workforce management (S)	Partnership with universities for Indigenous midwives and Aboriginal people, regional maternity care workers and increased Indigenous midwives, quality supervision, supervised GP training, professional development program attitudinal training attracted and enhanced the retention of the GPs.	Administrative burden, direct remuneration and opportunity costs (e.g., GP services in RACFs), poor trust and burnout, tension of roles of the workforce, inequitable distribution, lack of skills and experience, shortages of staff and high turnover, complex and overwhelming workload, a lack of understanding of administrative management functions, and inadequate skill experience.	[13, 27, 48, 57, 59, 60, 62, 71, 73, 75, 80, 82–85, 96, 105, 110–117, 133, 148, 154]
Evidence use in planning and monitoring (S)	Standard setting, benchmarking using reliable and comparable data, tailoring and prioritising practices, monitoring and surveillance, priority-based resource allocation in planning, timely collection of consistent and quality data from multiple sources and collation, creating visual indicators and prioritising, board training, accreditation requirements, research, practitioner-informed implementation and national quality and performance framework, tailored community-driven, bottom-up health planning using codesign principles, surveillance, reporting, use of records of care and quality indicators.	Inadequate quality data, poor understanding sensitivity in surveillance, complexity of use of technology, lack of comparable information, lack of audit tools and data on quality of care, inadequate evidence-based planning, missing link of translation of evidence into practice, unavailability of quality data and inadequacy to identify problems, poor coordination of health planning, inadequate funding of PHNs, performance and regulatory requirements.	[13, 15, 30, 46, 51, 55, 60–62, 70, 73, 76, 85–89, 96, 98, 106, 107, 113, 114, 117–120, 127, 136, 137, 143, 144, 148, 153, 154]

Distal level			
Socioeconomic and diversities (S and D)	Provision of Medicare	Lack of full-time work, economic hardship, systematic racial discrimination, dominant culture at HFs that inadequately captured Indigenous knowledge, values and culture, experiences of stigma and discrimination in employment.	[6, 15, 30, 63, 64, 66, 72, 79, 84, 96, 102, 104, 109, 130, 137, 138, 140, 142]

S: supply side; D: demand side.

## Data Availability

All data generated or analysed during this study are included in this published article (and its supplementary information files).

## Supplementary Materials

Supplementary Materials Table S1: Preferred Reporting Items for Systematic Reviews and Meta-Analyses extension for scoping reviews (PRISMA-ScR). Table S2: search domains and terms to identify the database records. Table S3: data-charting template to extract information from the included studies in the review.
